# Cortico–Cortical Paired Associative Stimulation (ccPAS) in Ageing and Alzheimer’s Disease: A Quali-Quantitative Approach to Potential Therapeutic Mechanisms and Applications

**DOI:** 10.3390/brainsci15030237

**Published:** 2025-02-24

**Authors:** Chiara Di Fazio, Marco Tamietto, Mario Stanziano, Anna Nigri, Eugenio Scaliti, Sara Palermo

**Affiliations:** 1 Department of Psychology, University of Turin, 10124 Turin, Italy; chiara.difazio@unito.it; 2 International School of Advanced Studies, University of Camerino, 62032 Camerino, Italy; 3 Department of Medical and Clinical Psychology, Tilburg University, 5037 AB Tilburg, The Netherlands; 4 Neuroradiology Unit, Diagnostic and Technology Department, Fondazione Istituto di Ricovero e Cura a Carattere Scientifico (IRCCS) Istituto Neurologico Carlo Besta, 20133 Milan, Italy; 5 ALS Centre, “Rita Levi Montalcini” Department of Neuroscience, University of Turin, 10124 Turin, Italy; 6 Human Science and Technologies, University of Turin, 10124 Turin, Italy; eugenio.scaliti@unito.it; 7 Department of Management “Valter Cantino”, University of Turin, 10124 Turin, Italy

**Keywords:** brain ageing, Alzheimer’s disease, cortical excitability, cortico–cortical spike timing-dependent plasticity, NIBS, ccPAS, motor performance, brain plasticity, neuromodulation

## Abstract

**Background/Objectives**: Cognitive decline and Alzheimer’s disease (AD) pose a major challenge for the ageing population, with impaired synaptic plasticity playing a central role in their pathophysiology. This article explores the hypothesis that cortico–cortical paired associative stimulation (ccPAS), a non-invasive brain stimulation technique, can restore synaptic function by targeting impaired spike-timing-dependent plasticity (STDP), a key mechanism disrupted in AD. **Methods**: We reviewed existing studies investigating the effects of ccPAS on neuroplasticity in both ageing and AD populations. **Results**: Findings suggest age-specific effects, with ccPAS improving motor performance in young adults but showing limited efficacy in older adults, likely due to age-related declines in synaptic plasticity and cortical excitability. In AD, ccPAS studies reveal significant impairments in long-term potentiation (LTP)-like plasticity, while long-term depression (LTD)-like mechanisms appear relatively preserved, emphasising the need for targeted neuromodulation approaches. **Conclusions**: Despite promising preliminary results, evidence remains limited and largely focused on motor function, with the impact of ccPAS on cognitive domains still underexplored. To bridge this gap, future research should focus on larger and more diverse cohorts to optimise ccPAS protocols for ageing and AD populations and investigate its potential for enhancing cognitive function. By refining stimulation parameters and integrating neuroimageing-based personalisation strategies, ccPAS may represent a novel therapeutic approach for mitigating neuroplasticity deficits in ageing and neurodegenerative conditions.

## 1. Introduction

The ageing global population is witnessing a surge in neurodegenerative conditions, with Alzheimer’s disease (AD) at the forefront [1,2,3,4]. Synaptic dysfunction is increasingly recognised as a primary contributor to AD pathogenesis, complementing the earlier amyloid- and tau-centric models. This shift underscores the critical role of synaptic health in the progression of AD, particularly the disruption of synaptic plasticity mechanisms like spike-timing-dependent plasticity (STDP). We hypothesise that the disruption of STDP mechanisms in AD significantly contributes to cognitive impairment and memory loss, as these disruptions prevent synapses from properly adjusting to neuronal activity patterns. Targeting this disruption through cortico–cortical paired associative stimulation (ccPAS) could restore synaptic function by re-synchronising neuronal firing patterns and re-establishing STDP mechanisms. Addressing synaptic deficits offers an avenue for innovative therapeutic interventions targeting early physiological changes and preserving brain function [5].

Spike-timing-dependent plasticity is a fundamental mechanism of synaptic plasticity, linking the timing of neuronal activity to the encoding of memories. In AD, STDP mechanisms are disrupted due to pathological changes in synaptic function, which impair memory encoding and retrieval. This impairment in STDP underlies many cognitive deficits observed in AD, including memory loss and the inability to effectively encode new information [6]. This cognitive impairment is exacerbated by the pathological accumulation of amyloid plaques and neurofibrillary tangles, the atrophy of critical brain regions such as the hippocampus, and large-scale damage to neuronal networks involved in memory processing [5,6,7,8,9]. Thus, we predict that restoring STDP through ccPAS will help re-synchronise neuronal activity, enhancing cognitive performance and memory in AD patients.

Emerging evidence suggests that non-invasive brain stimulation (NIBS) techniques, such as transcranial magnetic stimulation (TMS) and cortico–cortical paired associative stimulation (ccPAS), may restore synaptic plasticity [10,11,12,13]. These approaches target specific brain regions and modulate their activity to counteract synaptic dysfunction. Among these techniques, ccPAS shows particular promise in enhancing connectivity within cortical circuits, potentially improving motor and cognitive outcomes [13,14].

This hypothesis investigates the consequences of altered STDP mechanisms in AD and explores ccPAS as a tool to restore synaptic function. By integrating findings from three key studies, we aim to elucidate the potential of ccPAS in ageing and AD populations, identify research gaps, and propose future directions for clinical applications. Testable predictions include improvements in motor and cognitive function following ccPAS intervention, particularly in areas directly related to memory and learning, as well as greater efficacy in older people or those in the early stages of AD.

**Figure 1:**
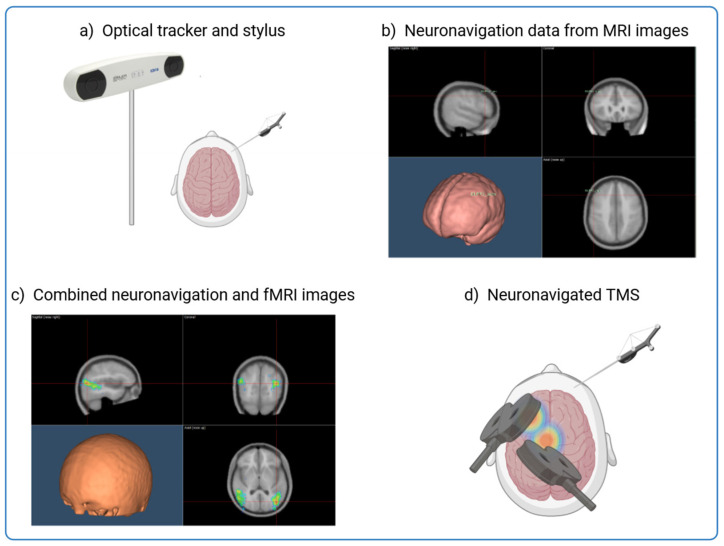
Integrating MRI and fMRI Data for Neuronavigation in TMS Procedures. The figure shows the process of combining structural MRI data with individual fMRI activation maps to guide the precise application of TMS to targeted brain regions. Neuronavigation aligns an MRI scan with the head of the subject through an alignment procedure and allows real-time tracking of the TMS coil and the head of the patient. This allows accurate guidance of the TMS coil to the intended neuroanatomical structure with millimeter precision. (**a**) The subject’s head is co-registered with the individual high-resolution anatomical MR image by utilising anatomical landmarks such as the nasion and crus helicis of the ears. (**b**) Creation of an individualised probabilistic brain model using the subject’s MRI images or by adapting resonance images from a template for each subject through a 3D warping procedure. (**c**) Neuronavigation process overlaying the fMRI activation onto the structural MRI to identify the optimal brain target for TMS. (**d**) Application of TMS to the identified brain regions.

TMS is central to this investigation as it provides a non-invasive method to assess and modulate cortical activity. However, its precision depends on the ability to precisely localise the targeted brain regions. Figure 1 shows how neuronavigation integrates individual MRI and fMRI data to precisely guide TMS application and ensure optimal stimulation of affected cortical areas in Alzheimer’s patients. By using anatomical landmarks and overlaying functional activation maps, this approach enables millimetre-precise tracking and alignment of the TMS coil in real time, improving the reliability and reproducibility of neuromodulation strategies [15,16,17,18,19,20] (Figure 1). This process is particularly valuable in AD, where cortical atrophy and individual variability in neurodegeneration patterns require personalised stimulation protocols to maximise therapeutic efficacy.

The integration of neuronavigation into ccPAS applications enhances its therapeutic precision. By tailoring stimulation to individual anatomical and functional characteristics, this method maximises the efficacy of plasticity induction and provides a personalised intervention strategy. Recent advancements in ccPAS protocols further support its potential to improve cognitive and motor functions by modulating targeted cortical circuits [21,22]. By testing the effects of personalised ccPAS in the few previous published clinical trials, we aim to validate the effectiveness of this intervention in both restoring STDP and enhancing cognitive resilience in AD patients.

**Figure 2:**
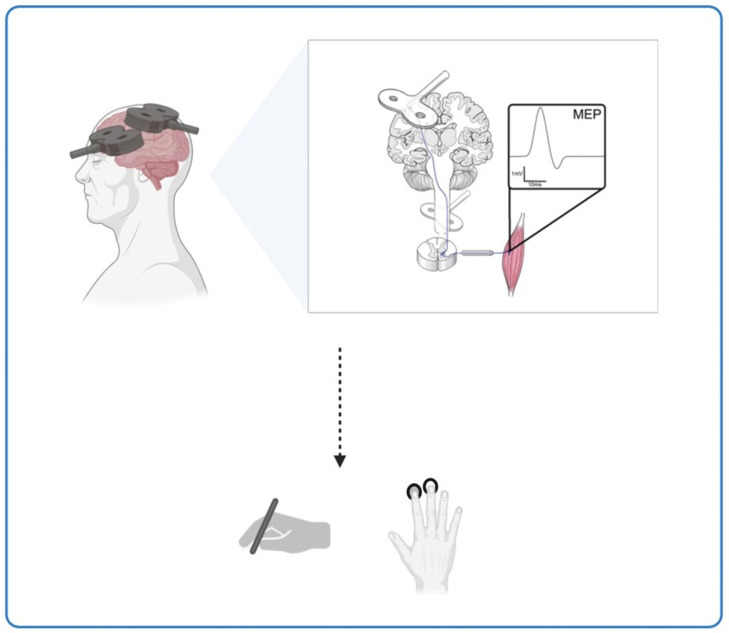
The potential of ccPAS stimulation in enhancing motor performance in Alzheimer’s disease and ageing. This figure illustrates the potential impact of ccPAS stimulation on cortical plasticity and motor performance across different age groups. It shows the effects on corticomotor excitability and fine motor skills, like hand dexterity, induced by ccPAS in the elderly, suggesting that this stimulation shows potential for enhancing motor performance and conserving neuroplasticity in the ageing population.

Beyond AD, this hypothesis has broader implications for ageing populations. Progressive neuronal dysfunction with age reduces plasticity, affecting motor skills such as manual dexterity and speed. ccPAS offers a promising avenue for rehabilitation, as it has been shown to modulate corticomotor excitability and enhance fine motor skills, particularly in elderly individuals who experience plasticity decline. Figure 2 illustrates the effects of ccPAS stimulation on cortical plasticity and motor function, emphasising its potential to counteract age-related neuroplasticity deficits. These findings suggest that ccPAS may not only mitigate age-related declines in motor performance but also provide a neuroprotective effect that could be leveraged for early intervention in neurodegenerative conditions (Figure 2).

## 2. Methods: A Quali-Quantitative Approach

To substantiate our hypothesis that cortico–cortical paired associative stimulation (ccPAS) can enhance cortical plasticity and improve brain reserve and resilience in brain ageing, we conducted a quali-quantitative review of the existing literature. This approach allowed us to provide a comprehensive synthesis of the current state of knowledge, integrating both qualitative insights and quantitative findings where possible, while identifying gaps that future research could address.

### 2.1. Literature Search

We utilised PubMed (https://pubmed.ncbi.nlm.nih.gov/ accessed on 15 December 2024) to identify studies exploring the potential of ccPAS in mitigating age-related declines in neuronal plasticity and its applications in rehabilitation for ageing and Alzheimer’s disease. The search strategy employed a Boolean query: [(ccPAS OR “cortico–cortical associative stimulation”) AND (“ageing” OR “Alzheimer’s disease” OR AD)]. Articles published between 2013 [23] and December 2024 were included.

### 2.2. Study Selection

The search yielded five articles, including four original research studies [24,25,26,27] and one systematic review [28]. Of those, the study by Momi et al. [27] was excluded as it did not involve the clinical populations of interest. The remaining articles were analysed to extract insights relevant to our hypothesis., focusing on ccPAS’s effects on neuronal plasticity, motor performance, and potential cognitive benefits.

**Figure 3:**
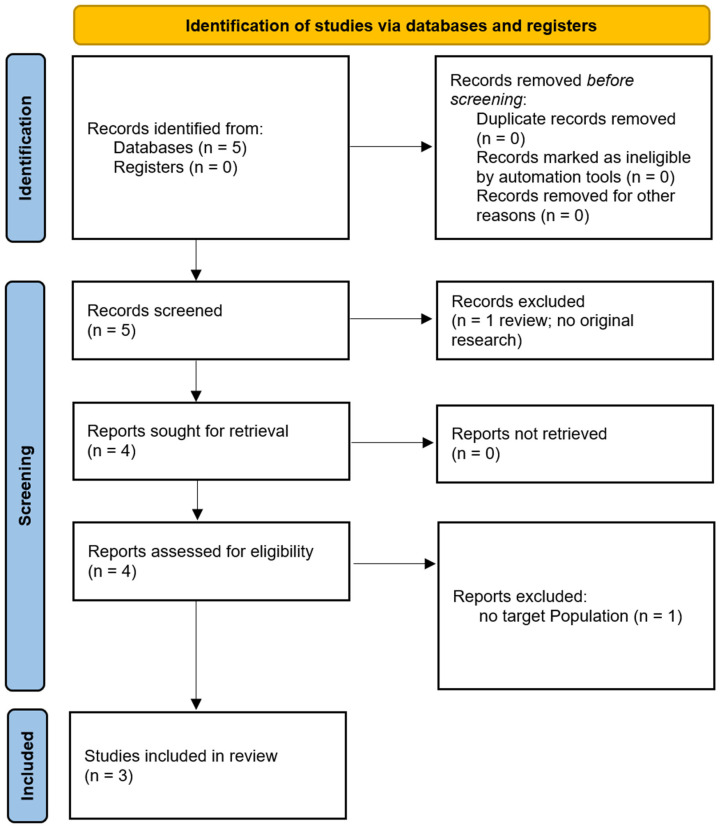
PRISMA flow diagram.

We have included a PRISMA flow diagram to illustrate the study selection process: one for the population selection and another for the treatment selection (see Figure 3). Additionally, we have summarised key details of the studies in tables. A brief overview of the populations studied is provided in Table 1, a summary of the ccPAS treatment parameters is provided in Table 2, and a summary of the major treatment outcomes for each study is provided in Table 3 [24,25,26].

### 2.3. Rationale for the Quali-Quantitative Approach

Given the limited number of studies available, it may not have been possible to conduct a full systematic review. With such a small body of literature, a systematic review might not have produced a meaningful or statistically robust synthesis of the evidence (e.g., insufficient sample size; high risk of bias; lack of statistical significance; risk of over-interpretation). Therefore, we opted for a qualitative-quantitative approach to integrate both qualitative findings and quantitative data from the selected studies. This approach allows contextualisation of the existing evidence and provides an initial overview of the potential therapeutic effects of ccPAS while acknowledging the significant gaps and inconsistencies that exist in the current research.

## 3. Main Findings

Study results provide valuable insight into the potential of ccPAS in modulating cortical plasticity. In these studies, neurophysiological and motor responses were assessed to paired associative stimulation protocols in different populations. Turrini et al. [24,25] studied healthy elderly individuals (MMSE: 27.1 ± 0.2 and 27.3 ± 2.1, respectively) with preserved cognitive and motor abilities. In contrast, Di Lorenzo et al. [26] focused on patients diagnosed with Alzheimer’s disease (AD), who were characterised by mild to moderate cognitive impairment (MMSE: 21.83 ± 2.7) and functional decline. The sample size was between 14 and 15 participants per group, with right-handed participants in the majority. Notably, older adults showed higher motor thresholds and lower motor dexterity than younger adults at baseline. This was observed in the 9-Hole Peg Test (9HPT) and the reaction time selection tasks (cRT).

The experimental protocols utilised a cortico–cortical paired associative stimulation (ccPAS) approach targeting different brain regions. Turrini et al. [24,25] stimulated the ventral premotor cortex (PMv) and M1 with an ISI of 8 ms, while Di Lorenzo et al. [26] applied ccPAS to the posterior parietal cortex (PPC) and primary motor cortex (M1) with interstimulus intervals (ISI) of ±5 ms. The stimulation frequency ranged from 0.1 Hz to 0.2 Hz and delivered 90–100 paired pulses. The intensity of PMv/PPC stimulation was set to 90% of the resting motor threshold (rMT), while M1 stimulation was adjusted to elicit motor-evoked potentials (MEPs) of ~1 mV. MEP amplitude served as the primary neurophysiological outcome, while behavioural assessment included 9HPT and cRT. Measurement time points varied between trials and captured both the immediate and delayed effects of ccPAS.

Turrini et al. [24] demonstrated that ccPAS improved performance on the 9HPT in young adults, but not in older adults. Furthermore, MEP modulation after ccPAS was a strong predictor of improvement in manual dexterity. ccPAS effects are age-dependent, which emphasises the need to adapt stimulation protocols to the physiological changes associated with ageing.

Building on these findings, Turrini et al. [25] investigated the relationship between neuroplasticity and motor performance in different age groups. The study found significant differences in baseline motor performance, with younger participants performing better than older adults on both the 9HPT and cRT tasks. During the ccPAS, young adults showed a progressive increase in corticospinal excitability, while older adults showed inconsistent MEP modulation. These results suggest that the efficacy of ccPAS in improving motor function is closely linked to preserved plasticity within the premotor–motor pathways.

Di Lorenzo et al. [26] extended the study to a pathological ageing context and investigated cortico–cortical STDP mechanisms in AD patients. The study revealed significant impairments in LTP-like plasticity after ccPAS between the PPC and M1, while LTD-like plasticity remained relatively intact. The results highlight the importance of synaptic dysfunction in AD and suggest that synaptic deficits contribute to cognitive decline. In addition, the study is consistent with previous research indicating that impaired plasticity is an early event in the neurodegenerative cascade leading to Alzheimer’s disease.

As a whole, these studies provide a basic understanding of ccPAS applications. They also highlight critical gaps, such as the limited evidence in Alzheimer’s populations and the need for larger, more diverse cohorts to validate results. Methodological differences highlight the multiple applications of ccPAS in ageing and neurodegenerative diseases, as well as the method’s potential to study changes in plasticity during healthy ageing and disease.

## 4. Discussion

Neuroplasticity, the brain’s ability to adapt and reorganise in response to experience, declines with age, contributing to functional decline and increasing the risk of neurodegenerative diseases such as Alzheimer’s disease (AD) [25].

As synaptic plasticity underlies many of the cognitive and motor functions that are impaired in AD, this emphasises the need for therapeutic approaches that target the underlying synaptic dysfunction. One of the most important pathophysiological changes in AD is the disruption of synaptic plasticity mechanisms, in particular spike-timing-dependent plasticity (STDP). In healthy people, STDP plays a crucial role in learning and memory by strengthening or weakening synaptic connections based on the temporal pattern of neuronal firing. In AD, however, the delicate balance between synaptic potentiation and depression is disrupted, leading to impaired memory consolidation and cognitive decline. We hypothesise that the disruption of STDP mechanisms in AD contributes directly to the cognitive deficits observed in the disease, as synaptic adjustments no longer align properly with neuronal activity patterns. This synaptic dysfunction is a central feature of AD that cannot be adequately explained by the accumulation of amyloid and tau alone. This emphasises the importance of considering synaptic plasticity mechanisms such as STDP to fully understand disease progression and develop therapeutic strategies targeting these early disruptions.

In light of these findings, NIBS techniques such as ccPAS have emerged as a promising approach to counteract synaptic dysfunction in AD [27,28,29]. By modulating STDP and improving synaptic plasticity, ccPAS offers a potential strategy to restore lost cognitive function. The efficacy of ccPAS lies in its ability to act on impaired STDP mechanisms in AD, potentially restoring the balance required for optimal synaptic function. Although studies to date are limited, our results support the hypothesis that ccPAS can restore cortical plasticity and improve cognitive and motor outcomes by directly modulating the signalling pathways disrupted by AD.

### 4.1. Differential Impacts of LTP and LTD in Ageing and Alzheimer’s Disease and Implications for ccPAS Protocols

The studies reviewed suggest age-specific effects of ccPAS, with younger adults showing greater improvement in plasticity and motor performance than older adults [24,25,26]. Age-related differences in the efficacy of ccPAS may be attributed to several neurobiological factors that influence synaptic plasticity. First, ageing naturally leads to a decline in STDP mechanisms due to changes in neurotransmitter systems, such as reduced levels of dopamine and acetylcholine, alterations in calcium homeostasis, and shifts in neuronal membrane properties [30,31]. These factors collectively diminish the brain’s capacity to undergo plastic changes, which could explain why older adults exhibit less pronounced benefits from ccPAS compared to younger individuals. Additionally, age-related reductions in CE, as highlighted by Palermo et al. [32], are closely linked to cognitive health and may further dampen the brain’s responsiveness to plasticity-inducing interventions like ccPAS. Furthermore, the accumulation of neuroinflammation and oxidative stress with age can impair synaptic function [33,34,35], potentially blunting the effects of ccPAS. Importantly, the differential impact of LTP and LTD mechanisms in ageing compared to AD suggests that ccPAS protocols may need to be tailored accordingly. While both ageing and AD are characterised by LTP impairments, the underlying mechanisms and severity differ, necessitating age-specific approaches to optimise ccPAS outcomes. In older adults, personalised ccPAS protocols, possibly complemented by neuronavigation, could improve therapeutic efficacy by considering individual neurophysiological profiles [36,37].

In AD, the synaptic dysfunction is more pronounced, in particular the disruption of the mechanisms of LTP, which are crucial for memory consolidation. LTP is typically disrupted in AD due to altered intracellular signalling, neurotransmitter dysfunction, and pathological accumulation of amyloid plaques and tau tangles, all of which impair synaptic efficiency. Interestingly, LTD is relatively well preserved in the early stages of AD, possibly because it is less dependent on the same molecular signalling pathways that enable LTP. This imbalance between impaired LTP and relatively preserved LTD may underlie the cognitive deficits seen in Alzheimer’s patients, as LTP plays an important role in strengthening synaptic connections for memory formation, while LTD is involved in synaptic pruning and memory refinement. This synaptic imbalance could contribute significantly to the memory and motor impairments observed in AD.

Given this imbalance between LTP and LTD, we propose that ccPAS protocols could be specifically tailored to restore LTP-like plasticity in AD patients. By restoring the proper timing for synaptic potentiation, ccPAS could help alleviate the cognitive deficits resulting from impaired LTP while preserving still-functioning LTD processes. This approach would specifically target the disturbances in STDP mechanisms that underlie the synaptic imbalance observed in Alzheimer’s disease.

Assuming these differential effects on synaptic plasticity, ccPAS could be tailored to specifically target LTP-like plasticity in Alzheimer’s patients, restoring cognitive and motor function by redressing the imbalance between LTP and LTD. The ability to personalise ccPAS protocols using advanced imaging techniques such as functional and structural MRI enables precise targeting of the affected cortical circuits. To further refine early intervention strategies, the identification of biomarkers that can detect Alzheimer’s disease at its earliest stages is crucial for optimising ccPAS use [38]. Biomarkers play a fundamental role in detecting synaptic dysfunction before significant neurodegeneration occurs. In this context, non-invasive biomarkers are particularly advantageous as they lend themselves to large-scale screening. Among these biomarkers, blood-based biomarkers have shown promise in detecting early synaptic dysfunction in Alzheimer’s disease. Recent studies have emphasised the potential of phosphorylated tau (p-tau) and neurofilament light (NfL) in plasma as reliable indicators of neurodegenerative disease progression [39]. In addition, neurophysiological markers derived from NIBS techniques, such as TMS-derived measures of cortical excitability, may provide insights into the integrity of synaptic plasticity mechanisms [40].

### 4.2. Incorporating Findings from Other Disease Models

In addition to studies focusing on AD, research on other pathological conditions has highlighted similar beneficial effects of paired associative stimulation on synaptic plasticity. For example, studies in stroke have shown that PAS can improve motor performance and cortical plasticity by targeting specific neuronal circuits [41]. This provides valuable insight into how PAS could be used to restore lost function in diseases such as Alzheimer’s, which are characterised by impaired synaptic plasticity. These results emphasise the potential of PAS not only in improving motor recovery but also in facilitating neuroplasticity in neurodegenerative diseases such as Alzheimer’s [41].

The common mechanisms observed in different neurodegenerative diseases support the hypothesis that PAS may act on common neuroplasticity pathways such as synaptic potentiation and depression with disease-specific adaptations. Research on stroke suggests that PAS modulates corticospinal excitability and affects both motor performance and neurophysiological traits, suggesting that it may improve recovery of motor function in diseases such as AD. However, these effects appear to be contradictory, as some studies have not found a clear correlation between neurophysiological changes and motor improvements. Similar differences in outcomes are to be expected in AD, where different challenges related to synaptic plasticity may require tailored PAS protocols to optimise outcomes.

New findings suggest that PAS may also be used in fronto-temporal dementia (FTD). In contrast to Alzheimer’s disease, FTD is characterised by considerable phenotypic and neuropathological variability, which makes early diagnosis and targeted interventions difficult [42,43]. However, despite this heterogeneity, some FTD subtypes exhibit residual neuroplasticity that could be modulated by NIBS techniques, including PAS [44]. Although studies on PAS in FTD are still limited, recent evidence suggests that stimulation targeting frontotemporal networks could improve cognitive and behavioural functioning in selected patients.

These findings from other neurodegenerative disease models contribute to our understanding of the broader applicability of ccPAS and its potential as a therapeutic tool for AD, particularly when considering the disease-specific neuroplasticity mechanisms that may influence its efficacy. By contextualising the role of ccPAS in stroke, we can refine the approach in AD and ensure that PAS protocols are better tailored to the unique neurophysiological and motor challenges of AD.

### 4.3. The Rationale for Specific ccPAS Parameters

The selection of ccPAS parameters in this perspective is based on findings from previous studies that examined both healthy and clinical populations (see Table 1). Interstimulus intervals (ISIs) of ±5 ms and ±8 ms have been shown to effectively modulate STDP by aligning with the natural temporal dynamics of neuronal firing [45,46,47,48]. These ISIs are critical for facilitating LTP, which is impaired in AD due to the loss of synapses and altered neurotransmitter signalling.

Stimulation frequencies of 0.1 to 0.2 Hz have been widely used in cortico–cortical connectivity studies and have been shown to be effective in improving plasticity without causing overstimulation [49,50]. This is particularly important in AD, where neuronal circuits are prone to excitotoxicity. The intensity of 90% of the resting motor threshold (RMT) was chosen to ensure sufficient activation of cortical circuits while minimising the risk of overstimulation, which is confirmed by previous results in neurodegenerative diseases [51].

The potential interactions between ccPAS and AD-related changes—such as cholinergic deficits, impaired calcium homeostasis, and increased neuroinflammation—underline the need for careful parameter optimisation [52,53,54]. Tailoring ccPAS protocols based on these factors could improve therapeutic outcomes and make ccPAS a viable intervention for cognitive and motor impairment in AD.

### 4.4. Challenges to Be Solved

Despite its potential, several challenges need to be overcome before ccPAS can be widely used in the clinical setting. One important limitation is the small sample size of the existing studies, which limits the generalisability of the results. In addition, the age-specific responses to ccPAS raise questions about its applicability beyond motor performance, particularly in broader cognitive domains. The translation of ccPAS from research to clinical practise is also associated with logistical and resource-related obstacles, including the need for specialised equipment, individualised protocols, and trained staff. In contrast to conventional TMS, ccPAS requires precise targeting of cortical circuits. This precision is often possible through the use of functional MRI or neuronavigation, which can significantly improve the targeting accuracy of stimulation sites through real-time mapping of individual brain regions.

The cost of repeated ccPAS sessions further complicates feasibility, especially when multiple sessions are required for sustained neuroplastic effects. Future research should focus on optimising protocol efficiency and developing cost-effective strategies to enable wider accessibility. This includes determining the minimum number of sessions required for clinically meaningful changes in cortical excitability (CE) and exploring ways to streamline the process without compromising treatment efficacy.

In addition to logistical challenges, patient-specific factors can also significantly influence the efficacy of ccPAS. The stage of AD progression is particularly important, as people in the early stages of AD may have greater neuroplastic potential than those with advanced neurodegeneration, where extensive synaptic loss may limit responsiveness. Genetic predispositions such as APOE ε4 status may also impact treatment outcomes, as it is associated with faster cognitive decline and increased synaptic vulnerability. In addition, comorbidities such as vascular dysfunction, which often accompanies AD, could influence the efficacy of ccPAS by altering CE and neurovascular coupling.

Moreover, addressing potential sources of side effects—including vagal alterations—is essential to ensure both the safety and optimal outcomes for the patient.

Tailoring ccPAS to the unique characteristics of each patient will maximise the chances of successfully activating the relevant neural circuits while minimising the risks associated with treatment and variability in response to treatment.

While current evidence primarily supports the effects of ccPAS on motor function, its potential impact on cognitive processes remains largely unexplored. The available studies have predominantly investigated ccPAS-induced plasticity within motor circuits, demonstrating improvements in motor performance and cortical excitability. However, the same neurophysiological principles underlying these effects suggest that ccPAS could also modulate higher-order cognitive networks, particularly those involved in memory and executive function. Given the differential neural substrates involved, it is crucial to distinguish between the mechanisms by which ccPAS influences motor function—primarily through modulation of sensorimotor connectivity—and cognitive function, which would likely involve stimulation of frontoparietal and hippocampal-related circuits. Future studies should focus on systematically assessing these cognitive effects, optimising stimulation parameters for non-motor brain regions, and developing task-specific paradigms to evaluate cognitive improvements in ageing and AD. Such research would pave the way for broader therapeutic applications, with the goal of enhancing both motor and cognitive outcomes.

### 4.5. Future Directions

To optimise the clinical application of ccPAS in AD and ageing populations, future studies need to address several key areas. First, it is imperative to increase sample size by conducting large-scale, powered studies that include diverse populations at different stages of AD. Such studies would allow the exploration of individual differences in factors such as age, disease severity, and cognitive function, leading to a better understanding of how ccPAS might benefit specific subgroups of patients.

Studies should prioritise the definition of specific outcome measures. These outcomes should focus on precise, standardised assessments that can either support or challenge the hypothesis about the potential benefits of ccPAS. Cognitive assessments are critical for evaluating the impact of ccPAS on domains known to be impaired in AD, such as memory, executive function, and attention [55,56]. Standardised tests such as the Mini-Mental State Examination or the Montreal Cognitive Assessment can assess general cognitive function, while domain-specific tests—such as the Rey Auditory Verbal Learning Test for memory and the Trail Making Test for executive function [6]—can provide more detailed insights into the impact of ccPAS.

In addition, neurophysiological measurements, including motor-evoked potentials generated by TMS and electroencephalograms, could be used to assess brain connectivity to track changes in CE and synaptic plasticity following ccPAS. These measurements would help to capture immediate changes in synaptic processes and provide direct evidence for the modulation of neuronal circuits involved in cognitive and motor tasks by ccPAS.

Exploring cognitive outcomes is another important component of future research. Studies should investigate the effects of ccPAS on different cognitive domains, particularly working memory, episodic memory, and executive function, all of which are strongly affected by AD. Longitudinal studies should be conducted to assess the durability of ccPAS-induced effects over time as well as their impact on real-world functional outcomes, such as activities of daily living and quality of life.

In terms of cerebral outcomes, future studies should investigate how ccPAS affects CE, LTP, and synaptic efficiency using advanced neuroimaging techniques. For example, fMRI and magnetic resonance spectroscopy could assess both structural and functional changes in the brain, particularly in regions involved in memory and executive function, such as the hippocampus, prefrontal cortex, and parietal regions. These cerebral biomarkers would provide direct evidence of how ccPAS alters synaptic processes at the macro and micro levels.

Neuronavigation should also be integrated into ccPAS protocols by combining structural and functional neuroimaging techniques with real-time targeting. Neuronavigation improves the precision of cortical targeting by tailoring the stimulation protocol to an individual’s unique brain anatomy, functional mapping, and craniometric data, optimising the chances of activating the relevant neural circuits. This approach would allow precise stimulation of cortical areas known to be affected by cognitive decline. Personalised ccPAS parameters, including stimulation intensity, frequency, and location, should be tailored to the individual’s specific neurophysiological profile, with a particular focus on areas such as the medial temporal lobe or the fronto-parietal network, which are particularly vulnerable in AD.

Multidisciplinary approaches are essential to further optimise the use of ccPAS in AD. Collaboration between fields such as neuroscience, neurology, and engineering will foster the development of customised, accessible ccPAS protocols. This collaboration is key to improving clinical applicability and patient adherence to ccPAS treatments, as well as integrating the latest advances in neuroimaging, neurostimulation, and clinical neuropsychology.

Further refinement of experimental designs is also required. Future studies should develop specific experimental parameters tailored to STDP impairment in AD. These include the incorporation of time intervals (e.g., varying the duration of short and long interval pairs in ccPAS protocols) and modulation of frequency to restore the precise timing required for optimal synaptic plasticity. In addition, protocols that aim to directly influence the balance between LTP and LTD, or those that adapt to an individual’s neurophysiological profile, could improve the efficacy of ccPAS.

By integrating these specific experimental designs and outcome measures, future research can provide a clearer, evidence-based framework for understanding ccPAS as a tool to improve synaptic plasticity and cognitive function in AD. Several testable predictions can be made:

- Whether ccPAS can induce measurable changes in LTP-like plasticity in key brain regions such as the hippocampus and prefrontal cortex.
- Whether such changes in synaptic plasticity correlate with improvements in memory tasks or real-world functional outcomes.
- The durability of ccPAS-induced improvements in motor and cognitive function and whether these effects can delay the progression of AD.

Recent work by Palermo et al. [32] emphasises the potential of cortical excitability (CE) as an indicator of cognitive health in ageing populations. Their perspective article emphasises that changes in CE could serve as a marker of resilience to cognitive decline in older adults and thus provide a non-invasive measure of healthy ageing. This concept is particularly relevant to ccPAS, as CE may reflect the very synaptic changes that ccPAS is designed to modulate. In addition, neurophysiological measurements, including motor-evoked potentials generated by TMS and electroencephalograms, could be used to assess brain connectivity to track changes in CE and synaptic plasticity after ccPAS. These measurements would help to capture immediate changes in synaptic processes and provide direct evidence for the modulation of neuronal circuits involved in cognitive and motor tasks by ccPAS. Furthermore, the integration of neuronavigation using structural resting-state MRI and fMRI data could refine ccPAS by precisely localising the affected brain regions to ensure precise stimulation and maximise efficacy. This personalised approach is a step towards precision medicine.

Palermo et al. also discusses the challenges associated with implementing CE as a standardised measure, including the need for consistent measurement protocols. As their work suggests, with proper consideration and integration, CE could not only aid in the early detection of cognitive decline but also enable targeted interventions to improve cognitive resilience. By using such biomarkers in future ccPAS studies, we can refine our approaches to personalised neurostimulation and potentially improve the efficacy of interventions aimed at mitigating the effects of ageing and AD.

### 4.6. Limitations

A key limitation of this manuscript is the inability to conduct a full systematic review due to the limited number of studies directly addressing the enhancement of cortical plasticity by ccPAS in brain ageing. While we aimed to provide a comprehensive overview, only three studies were identified, making a systematic review or meta-analysis impossible. Consequently, our approach remains a narrative overview with qualitative-quantitative synthesis. We recognise that this approach, while useful for contextualising the hypothesis and identifying research gaps, has limitations, particularly in terms of generalisability and methodological rigor. Future studies with a larger evidence base will be essential for a more systematic assessment and a deeper understanding of ccPAS effects on brain ageing.

## 5. Conclusions

Disruptions in synaptic plasticity and cortico–cortical connectivity are key factors in AD and contribute to cognitive deficits and memory impairment. Patients in the early stages of the disease often show increased brain oscillations and synchronisation between distant regions, indicating network dysfunction. ccPAS can induce neuroplasticity in specific areas and promote compensatory changes to restore normal neuronal function. NIBS is effective, safe, and associated with minimal side effects. It offers a promising therapeutic strategy to improve cognitive function and potentially delay disease progression.

Our hypothesis highlights the potential impact of restoring STDP on cortical plasticity and therapeutic outcomes. Integrating neuronavigation using resting-state structural MRI and fMRI data could refine ccPAS by precisely localising the affected brain regions, ensuring precise stimulation, and maximising efficacy. This personalised approach is a step towards precision medicine, revolutionising the treatment of neurodegenerative diseases and offering hope for better cognitive outcomes and independence.

## Figures and Tables

**Table 1:** Characteristics of the population in the included studies.

	Turrini et al. [24]	Turrini et al. [25]	Di Lorenzo et al. [26]
Sample Size	14	14	15
Age (Mean ± SD)	72 ± 6 years	71.21 ± 6.95 years	69.5 ± 6.8 years
Gender (Female %)	N/A	3/14 Female	53%
Education (Years, Mean ± SD)	N/A	N/A	8.3 ± 4.1
MMSE (Mean ± SD)	27.1 ± 0.2	27.3 ± 2.1	21.83 ± 2.7
Other Clinical Measures	MMSE corrected score 27.1 ± 0.2, Raven’s colored progressive matrices 29.6 ± 0.5, Adequate power grip and precision grip strengths	MMSE corrected score 27.3 ± 2.1, Raven’s colored progressive matrices 29.8 ± 4.8, Adequate power grip and precision grip strengths	CDR 0.8 ± 0.6; ADL 5.3 ± 0.6; IADL 7.3 ± 0.7
Inclusion Criteria	Healthy volunteers, right-handed, normal or corrected vision, MMSE score, grip strength	Right-handed, normal or corrected vision, MMSE score, grip strength, no contraindications to TMS	Clinical dementia criteria as defined by DSM-IV and typical Alzheimer’s disease according to the IWG criteria
Exclusion Criteria	Adverse reactions to TMS	Contraindications to TMS	Specific cognitive deficits, acute stroke, ischemic lesions, CSF Aβ1-42 values > 600 pg/mL, use of drugs modulating cortical excitability
Baseline 9HPT (Mean ± SD)	31 ± 7 s	30 ± 6 s	N/A
Baseline cRT (Mean ± SD)	N/A	587 ± 150 ms	N/A
rMT (Mean ± SD)	57 ± 17%	N/A	N/A

Note: N/A = Not Available in the source. Abbreviations: CDR = Clinical Dementia Rating scale; CSF = cerebrospinal fluid; DSM-IV = diagnostic and statistical manual of mental disorders—fourth revision; IWG = International Working Group; MMSE = Mini-Mental State Examination.

**Table 2:** ccPAS treatment parameters in the included studies.

	Turrini et al. [24]	Turrini et al. [25]	Di Lorenzo et al. [26]
Stimulation Protocol	ccPAS (PMv-to-M1)	ccPAS (PMv-to-M1)	ccPAS (PPC-to-M1)
ISI	8 ms (PMv precedes M1)	8 ms (PMv precedes M1)	+5 ms (PPC precedes M1) −5 ms (PPC follows M1)
Frequency	0.1 Hz	0.1 Hz	0.2 Hz
Number of Pulses/Pairs	90 pairs	90 pairs	100 pairs
Stimulation Intensity CS	90% of rMT	90% of rMT	90% of rMT
Stimulation Intensity TS	90% of rMT	90% of rMT	90% of rMT
Stimulation Intensity M1	Evoke MEP of ~1 mV	Evoke MEP of ~1 mV	Evoke MEP of ~1 mV
MEP Measurement	Amplitude	Amplitude	Amplitude
Tasks	9HPT, cRT	9HPT, cRT	N/A
Time Points for Measurement	Baseline, Pre, T0, T30	Baseline	Baseline, T0, T10, T20
Stimulation Coordinates	PMv x = −52 y = 10 z = 24	PMv x = −53.6 ± 2.0 y = 9.6 ± 1.5 z = 23.7 ± 1.1 M1 x = −33.6 ± 6.3 y = −18.6 ± 7.7 z = 59.7 ± 4.2	N/A

Note: N/A = Not Available in the source. Abbreviations: cRT = choice reaction task; CS = conditioning stimulus; M1 = primary motor cortex; MEP = motor-evoked potentials; ISI = Interstimulus Interval; PPC-to-M1 = posterior parietal cortex to primary motor cortex; PMv-to-M1 = ventral premotor cortex to primary motor cortex; rMT = resting motor threshold; T = timing; TS = test stimulus; 9HPT = 9-Hole Peg Test.

**Table 3:** Treatment effects and outcome measures.

	Turrini et al. [24]	Turrini et al. [25]	Di Lorenzo et al. [26]
MEP Amplitude	Reduced plasticity and reactivity vs. young adults.	Lower motor performance and PMv-M1 plasticity.	No significant MEP changes after ccPAS.
9HPT Performance	Older adults had lower dexterity than young adults (31 ± 7 s vs. 22 ± 2 s, *p* < 0.001). ccPAS improved performance less in older adults.	Higher rMT in older adults (*p* = 0.01). MEP slope predicted 9HPT performance (β = −0.67, *p* < 0.0001).	N/A
cRT Performance	Older adults had slower visuomotor speed than young adults (597 ± 139 ms vs. 392 ± 24 ms, *p* < 0.001). ccPAS was less effective.	MEP slope predicted cRT performance (β = −0.64, *p* < 0.0001).	N/A
Correlation Analysis	N/A	N/A	No link between MEP change and clinical factors (age, education, disease duration, MMSE, CSF biomarkers).
MEP Changes During ccPAS	N/A	No consistent MEP modulation in elderly (*p* = 0.14). MEP changes were smaller than in young participants (*p* = 0.013).	N/A

Note: *p* < 0.05 was considered significant in [26]; Statistical data from [24,25] are reported where available. N/A = Not Available in the source. Abbreviations: cRT = choice reaction task; CSF = cerebrospinal fluid; MEP = motor-evoked potentials; MMSE = Mini-Mental State Examination; 9HPT = 9-Hole Peg Test.

## Data Availability

No new data were created or analyzed in this study. Data sharing is not applicable to this article.
